# Association of N-terminal pro-B-type natriuretic peptide levels and mortality risk in acute myocardial infarction across body mass index categories: an observational cohort study

**DOI:** 10.1186/s13098-023-01163-1

**Published:** 2023-10-06

**Authors:** Man Wang, Ning Cao, Li Zhou, Wen Su, Hui Chen, Hongwei Li

**Affiliations:** 1 Department of Cardiology, Cardiovascular Centre, Beijing Friendship Hospital, Capital Medical University, No.95, Yongan Road, Xicheng District, Beijing, 100050 People’s Republic of China; 2 Beijing Key Laboratory of Metabolic Disorder Related Cardiovascular Disease, Beijing, China; 3 Laboratory for Clinical Medicine, Capital Medical University, Beijing, China

**Keywords:** N-terminal pro-B-type natriuretic peptide, Acute myocardial infarction, Body mass index, Mortality risk

## Abstract

**Background:**

The prognostic value of N-terminal pro-B-type natriuretic peptide (NT-proBNP) across body mass index (BMI) categories in patients with acute myocardial infarction (AMI) is unclear. We aimed to assess the predictive value of NT-proBNP levels and identify the best cutoff values for mortality risk prediction across BMI categories in AMI.

**Methods:**

We analyzed 4677 patients with AMI from the Cardiovascular Centre Beijing Friendship Hospital Database Bank. Patients were classified into underweight (< 18.5 kg/m^2^), normal-weight (18.5–23.9 kg/m^2^), overweight (24–27.9 kg/m^2^), and obese (≥ 28 kg/m^2^) groups. The association between NT-proBNP (ln-transformed) and mortality was investigated using Cox regression and stratified by BMI.

**Results:**

During follow-up (13,787 person-years of observation), 718 patients died, averaging 52.1 events per 1000 person-years. NT-proBNP levels were inversely correlated with BMI (*β* = − 0.096, *P* < 0.001). After adjustment, NT-proBNP was independently associated with all-cause mortality (hazard ratio [HR] per 1-SD: 1.82; 95% confidence interval [CI] 1.60–2.07) in patients with AMI. Similar findings were observed in analyses stratified by BMI category, except for the underweight group. Adding NT-proBNP to conventional risk models improved risk discrimination in normal-weight, overweight, and obese patients (C-index changes of 0.036, 0.042, and 0.032, respectively) and classification of patients into predicted mortality risk categories (net reclassification improvement 0.263, 0.204, and 0.197, respectively). The best NT-proBNP cutoff values for 5-year mortality risk prediction across BMI categories were 5710, 4492, 2253, and 1300 pg/ml.

**Conclusion:**

NT-proBNP level was an independent prognostic factor for mortality in patients with AMI and varied according to BMI. The best NT-proBNP cutoff values for mortality risk prediction reduced as BMI increased.

**Supplementary Information:**

The online version contains supplementary material available at 10.1186/s13098-023-01163-1.

## Introduction

Acute myocardial infarction (AMI) remains a major cause of morbidity and mortality worldwide despite improvements in clinical strategies over the past decade [1]. Early and precise risk stratification is essential in patients with AMI. Patients with a high mortality risk would benefit from intensive pharmacological treatments or early interventions.

N-terminal pro-B-type natriuretic peptide (NT-proBNP) is a useful marker of cardiac reserve and haemodynamic stress, which provides prognostic information [2]. Plasma NT-proBNP levels rise rapidly to a maximum at 20–30 h after the onset of symptoms due to acute left ventricular dysfunction during early AMI [3]. NT-proBNP is a strong independent predictor of mortality in patients with AMI [4–8]. However, the plasma concentrations of NT-proBNP are affected by various factors [9–11], such as age, sex, renal function, and body mass index (BMI).

An inverse relationship exists between NT-proBNP levels and BMI in patients with non-ischaemic disease [12–14]. In addition, NT-proBNP’s ability to predict risk in different BMI categories is contradictory in heart failure [12, 15, 16]. The relationship between NT-proBNP levels and BMI in patients with AMI remains unknown. Given the growing prevalence of obesity and its importance as a risk factor for cardiovascular diseases, it is vital to understand the potential effect of BMI on the predictive value of NT-proBNP levels across BMI categories in AMI.

Thus, we primarily aimed to evaluate the prognostic value of NT-proBNP levels in a large Chinese cohort of patients with AMI. The secondary aims of this study were: (i) to compare the utility of NT-proBNP for mortality risk prediction among patients with AMI in different BMI categories, and (ii) to determine the optimal NT-proBNP cutoff values for predicting mortality risk across BMI categories.

## Materials and methods

### Study population

The Cardiovascular Centre Beijing Friendship Hospital Database Bank (CBDBANK) consecutively collected the medical records of inpatients diagnosed with acute coronary syndrome (ACS) in the Department of Cardiology at the Beijing Friendship Hospital. A total of 15,330 patients with ACS were enrolled from January 2013 to January 2021, of which 4848 were diagnosed with AMI. We excluded 171 patients with missing BMI or NT-proBNP data, leaving 4677 patients with AMI in this study (Additional file 1). The study was approved by the Ethics Committee of Beijing Friendship Hospital, Capital Medical University (2021-P2-107-01) and written informed consent was obtained from all patients.

### Definition of AMI

AMI was defined as a typical rise and/or fall of cardiac troponin values with at least one value above the 99th percentile upper reference limit and at least one of the following: symptoms of AMI; new-onset significant ST-segment or T-wave change or left bundle branch block; development of a pathologic Q-wave in ≥ 2 contiguous electrocardiogram leads; imaging evidence of new viable myocardium loss or regional wall motion abnormality in a pattern consistent with an ischaemic aetiology; and identification of an intracoronary lesion on angiography [17]. Patients underwent standard medical and interventional management for AMI [18, 19]. Patients undergoing percutaneous coronary intervention (PCI) received heparin and loading doses of aspirin (300 mg) and clopidogrel (300–600 mg) (or 180 mg of ticagrelor). Standard therapies after PCI were continued at hospital discharge, including the maintenance dose of aspirin (100 mg/day), clopidogrel (75 mg/day) or ticagrelor (180 mg/day), statins, angiotensin-converting enzyme inhibitors (ACEI) or angiotensin II receptor blockers (ARB), and beta-blockers.

### Measurements of NT-proBNP levels

NT-proBNP levels were measured at the time of initial admission, as well as at 12, 24, 48, and 72 h, and 5 days after admission using a chemiluminescent enzyme immunoassay (PATHFAST Immunoanalyzer, PHC Europe B.V.). The variations assay coefficients range from 4.6% to 5.4%. The analytical range of the assay was 15–30,000 pg/ml. Peak NT-proBNP values were used in the analyses.

### Definition of the BMI categories

BMI was calculated as the ratio of weight in kilograms to the square of the height in meters. According to the Working Group on Obesity in China, patients were classified as: underweight, < 18.5 kg/m^2^; normal, 18.5–23.9 kg/m^2^; overweight, 24–27.9 kg/m^2^; and obese, ≥ 28 kg/m^2^ [20].

### Follow-up and outcome

Patient outcomes during hospitalisation were confirmed using their medical records. Clinical follow-up after discharge was performed at 1, 6, and 12 months and thereafter annually via telephonic interviews or medical records if patients visited the outpatient clinic. The primary endpoint was all-cause mortality during hospitalisation and follow-up. The second endpoint was cardiac death, which was defined as death caused by AMI or heart failure, or documented sudden cardiac death.

### Covariates

The following baseline characteristics were collected: demographic information (age, sex), lifestyle, medical history, laboratory results, and therapy. Lifestyle included smoking and drinking status (none, ever, and current). Medical history including hypertension, diabetes mellitus, dyslipidaemia, and previous coronary heart and chronic kidney diseases were self-reported. Overnight fasting blood samples were taken from the antecubital vein for routine biochemistry testing including low-density lipoprotein cholesterol (LDL-C), high-density lipoprotein cholesterol (HDL-C), total cholesterol, triglycerides, haemoglobin, fasting plasma glucose (FPG), high-sensitivity C-reactive protein (hs-CRP), high-sensitivity Troponin I (hs-TnI), and creatinine levels using standard methods. The estimated glomerular filtration rate (eGFR) was calculated using the Modification of Diet in Renal Disease (MDRD) equation [eGFR (mL/min/1.73 m^2^) = 175 × (serum creatinine)^−1.154^ × (age)^−0.203^ × (0.742 if female) × (1.212 if African American)] [21]. Left ventricular ejection fraction (LVEF) was assessed by expert cardiologists or certified sonographers on echocardiography using the Simpson’s method; LVEF ≤ 50% indicated left ventricular systolic dysfunction. Medication and intervention details, including aspirin, clopidogrel or ticagrelor, statins, β-blockers, ACEI or ARB, diuretics use and PCI were obtained directly from medical records.

We calculated the Global Registry of Acute Coronary Events (GRACE) risk score for long-term mortality with variables including age, heart rate, systolic blood pressure, initial serum creatinine levels, history of congestive heart failure, history of myocardial infarction, elevated cardiac marker levels, ST-segment depression, and absence of in-hospital PCI [22].

### Statistical analysis

Categorical variables are presented as frequencies (percentages). Continuous variables with normal distribution are reported as means and standard deviations (SD), whereas variables with skewed distribution are reported as median (interquartile range [IQR]). Participants in different BMI categories were compared using the chi-square or Fisher’s exact test for categorical variables and one-way analysis of variance or Kruskal–Wallis H test for continuous variables. Linear regression analysis was used to investigate the correlation between BMI and NT-proBNP levels (ln-transformed).

Univariate and multivariate Cox proportional hazard regression models were applied to identify the independent predictors of mortality. Hazard ratios (HRs) and 95% confidence intervals (CIs) were calculated. The following variables were assessed in the univariate analysis: sex, NT-proBNP levels, GRACE risk score, LVEF, BMI, history of hypertension and diabetes, hs-CRP levels, the peak hs-TnI value, and acute treatments (including aspirin, clopidogrel/ticagrelor, ACEI/ARB, β-blocker, statins, and diuretics). To minimise the risk of overfitting, we included only the GRACE risk score (per decile) in the regression models instead of its individual component. Variables with a skewed distribution (NT-proBNP, hs-CRP, and hs-TnI) were ln-transformed before being entered into the regression models; the risk estimations should be considered for each unitary increase in ln-value. Cox regression with backward stepwise selection was used to determine the variables to be included in the multivariate models.

We subsequently conducted stratified analyses to examine the association between NT-proBNP levels and mortality risk in different BMI categories. Based on the results of the multivariate Cox model, three models were used. Model 1 was unadjusted. Model 2 was adjusted for long-term GRACE risk score, and LVEF. Model 3 was adjusted for the covariates in Model 2 plus sex, diabetes, aspirin, clopidogrel/ticagrelor, and statin use.

To evaluate the impact of NT-proBNP levels on the mortality risk, the continuous net reclassification improvement (NRI), integrated discrimination improvement (IDI), and change in Harrell’s C-statistic were calculated by adding NT-proBNP levels to the existing clinical model (long-term GRACE risk score). We conducted a time-dependent receiver-operator curve (ROC) analysis and estimated the best NT-proBNP cutoffs levels for predicting the 5-year cardiac and all-cause mortality using Youden’s statistic for each BMI category.

Sensitivity analyses were performed by categorizing BMI as per the standard WHO criteria (underweight, < 18.5 kg/m^2^; normal weight, 18.5–24.9 kg/m^2^; overweight, 25–29.9 kg/m^2^; and obese, ≥ 30 kg/m^2^).

All analyses were performed using Stata (version 17.0; StataCorp, LP, College Station, TX, USA) and R (version 4.1.2; R Foundation for Statistical Computing). A two-sided *P*-value of < 0.05 was considered to be statistically significant. No adjustments were made for the multiple comparisons. Because of the potential type I errors that may occur with multiple comparisons, the findings should be interpreted as exploratory and only used to generate new hypotheses.

## Results

The cohort consisted of 4677 patients with AMI (mean age 64.7 years and 72.8% men). The mean BMI in the overall population was 25.4 ± 3.7 kg/m^2^, and 2.7% of patients were underweight (n = 127), 32.0% had normal weight (n = 1496), 43.4% were overweight (n = 2029), and 21.9% were obese (n = 1025). The baseline characteristics across BMI categories are summarised in Table 1. Patients with a higher BMI tended to be younger; were more likely men and current smokers; had a higher proportion of hypertension and dyslipidaemia; had higher mean levels of systolic blood pressure, FPG, haemoglobin, eGFR, LDL-C, total cholesterol, and triglycerides; and were more likely to use ACEI/ARB.

**Table 1:** Baseline characteristics according to the BMI categories

	Overall n = 4677	< 18.5 kg/m^2^ n = 127 (2.7%)	18.5–23.9 kg/m^2^ n = 1496 (32.0%)	24–27.9 kg/m^2^ n = 2029 (43.4%)	≥ 28 kg/m^2^ n = 1025 (21.9%)	*P* value
Clinical characteristics						
Age, years	64.7 (12.5)	75.4 (10.4)	67.7 (12.0)	63.9 (11.9)	60.7 (13.1)	< 0.001
Male, n (%)	3405 (72.8)	61 (48.0)	1037 (69.3)	1538 (75.8)	769 (75.0)	< 0.001
Hypertension, n (%)	3037 (64.9)	70 (55.1)	885 (59.2)	1335 (65.8)	747 (72.9)	< 0.001
Dyslipidemia, n (%)	2062 (44.1)	31 (24.4)	602 (40.2)	919 (45.3)	510 (49.8)	< 0.001
Diabetes, n (%)	1566 (33.5)	21 (16.5)	508 (34.0)	679 (33.5)	358 (34.9)	< 0.001
Prior MI, n (%)	514 (11.0)	18 (14.2)	168 (11.2)	235 (11.6)	93 (9.1)	0.11
Current smoker, n (%)	2114 (45.2)	44 (34.6)	630 (42.1)	954 (47.0)	486 (47.4)	< 0.001
STEMI, n (%)	2319 (49.6)	64 (50.4)	744 (49.7)	1031 (50.8)	480 (46.8)	0.22
Killip Class III-IV, n (%)	478 (10.2)	22 (17.3)	170 (11.4)	192 (9.5)	94 (9.2)	0.009
LM or multivessel disease, n (%)	3441 (73.6)	54 (42.5)	1053 (70.4)	1556 (76.7)	778 (75.9)	< 0.001
LVEF, %	58.1 (10.3)	55.5 (13.0)	57.4 (11.0)	58.3 (10.0)	58.7 (9.6)	< 0.001
Heart rate, bpm	75.5 (15.1)	77.3 (17.6)	75.8 (16.5)	75.1 (14.3)	75.4 (14.2)	0.28
SBP, mmHg	128.9 (22.3)	126.3 (23.8)	127.3 (22.6)	128.7 (21.9)	132.0 (22.1)	< 0.001
Long-term GRACE risk score	113.6 (27.7)	139.5 (26.1)	120.8 (27.1)	111.2 (26.0)	104.6 (27.6)	< 0.001
Laboratory values						
FPG, mmol/L	6.8 (2.8)	5.5 (2.0)	6.7 (2.9)	6.9 (2.8)	7.0 (2.8)	< 0.001
Hemoglobin, g/L	132.9 (22.5)	116.1 (23.2)	128.4 (22.5)	135.1 (21.9)	137.3 (21.4)	< 0.001
eGFR, mL/min/1.73m^2^	107.2 (36.5)	97.8 (47.1)	104.3 (37.9)	108.6 (35.4)	109.6 (34.8)	< 0.001
hs-CRP, mg/L	6.9 (2.3, 19.2)	10.2 (1.4, 19.8)	6.2 (1.8, 19.3)	6.8 (2.3, 19.0)	7.9 (2.8, 19.6)	0.010
Peak value of hs-TnI, ng/mL	7.6 (1.2, 35.7)	2.7 (0.6, 11.4)	7.3 (1.1, 34.8)	8.3 (1.3, 37.4)	7.6 (1.1, 34.7)	< 0.001
Peak value of NT-proBNP, pg/mL	1524 (491, 4787)	8974 (2348, 24123)	2064 (700, 6922)	1348 (455, 3892)	1041 (338, 3007)	< 0.001
LDL-C, mmol/L	2.6 (0.8)	2.2 (0.7)	2.5 (0.8)	2.6 (0.8)	2.7 (0.8)	< 0.001
HDL-C, mmol/L	1.0 (0.2)	1.2 (0.3)	1.1 (0.3)	1.0 (0.2)	1.0 (0.2)	< 0.001
Total cholesterol, mmol/L	4.5 (1.1)	4.1 (1.0)	4.3 (1.1)	4.5 (1.1)	4.6 (1.1)	< 0.001
Triglyceride, mmol/L	1.4 (1.0, 2.0)	0.9 (0.7, 1.2)	1.2 (0.9, 1.7)	1.5 (1.1, 2.1)	1.6 (1.2, 2.3)	< 0.001
Acute treatment						
Aspirin, n (%)	4181 (89.4)	92 (72.4)	1286 (86.0)	1862 (91.8)	941 (91.8)	< 0.001
Clopidogrel/Ticagrelor, n (%)	3895 (83.3)	82 (64.6)	1197 (80.0)	1747 (86.1)	869 (84.8)	< 0.001
ACEI/ARB, n (%)	2954 (63.2)	50 (39.4)	853 (57.0)	1327 (65.4)	724 (70.6)	< 0.001
β-blocker, n (%)	3356 (71.8)	74 (58.3)	1014 (67.8)	1512 (74.5)	756 (73.8)	< 0.001
Statins, n (%)	3989 (85.3)	89 (70.1)	1236 (82.6)	1773 (87.4)	891 (86.9)	< 0.001
Diuretics, n (%)	586 (12.5)	22 (17.3)	199 (13.3)	244 (12.0)	121 (11.8)	0.22
PCI, n (%)	3469 (74.2)	52 (40.9)	1037 (69.3)	1595 (78.6)	785 (76.6)	< 0.001

Values are n (%), mean (SD), or median (interquartile interval)

ACEI, angiotensin-converting enzyme inhibitor; ARB, angiotensin receptor blocker; BMI, body mass index; eGFR, estimated glomerular filtration rate; FPG, fasting plasma glucose; HDL-C, high-density lipoprotein cholesterol; hs-CRP, high sensitivity C-reactive protein; hs-TnI, high sensitivity troponin I; LDL-C, low-density lipoprotein cholesterol; LM, left main coronary artery; LVEF, left ventricular ejection fraction; MI, myocardial infarction; NT-proBNP, N-terminal pro-B-type natriuretic peptide; PCI, percutaneous coronary intervention; SBP, systolic blood pressure; STEMI, ST-segment elevated myocardial infarction

**Fig. 1:**
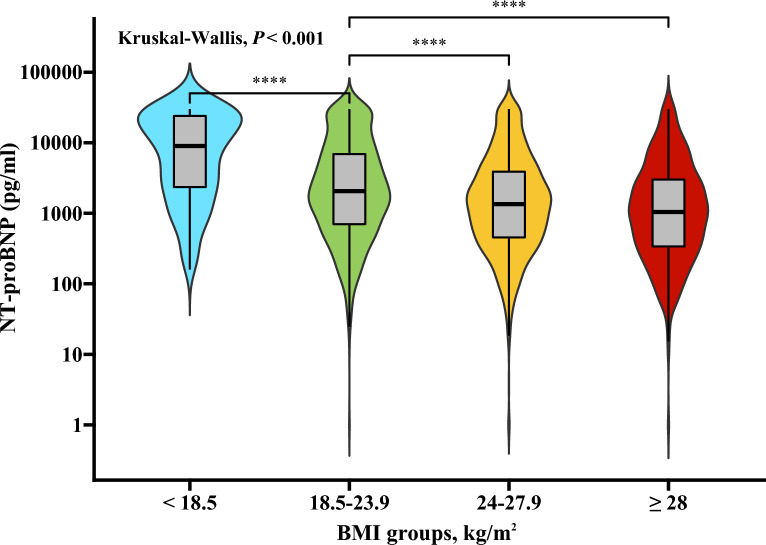
NT-proBNP concentrations across the BMI categories

The median NT-proBNP level in the overall population was 1524 pg/ml (IQR: 491–4787 pg/ml). NT-proBNP levels decreased as BMI increased (*β* = − 0.096, 95% CI − 0.108 to − 0.084; *P* < 0.001) (Fig. 1). The median NT-proBNP level was significantly higher in females than in males (3081 [IQR: 1155–9481] vs. 1160 [IQR: 412–3392] pg/ml; *P* < 0.001) and in patients aged ≥ 65 years than in those aged < 65 years (3223 [IQR: 1162–9115] vs. 811 [IQR: 311–1994] pg/ml; *P* < 0.001). The differences remained significant across the BMI categories (Additional file 2).

During follow-up (13,787 person-years of observation), 718 patients died, averaging 52.1 events per 1000 person-years. In total, 331 (7.1%) cardiac deaths were reported. The lower BMI category was associated with a higher all-cause mortality, with an incidence of 45.7%, 21.0%, 12.0%, and 10.0% in the underweight, normal-weight, overweight and obese groups, respectively (*P* < 0.001) (Additional file 3. Similarly, the incidence of cardiac death was also higher in the underweight group (19.7%), followed by the normal weight (9.4%), overweight (5.8%), and obese (4.8%) groups (*P* < 0.001) (Additional file 3).

Multivariate Cox regression analysis showed that the ln-transformed NT-proBNP levels were an independent predictor of all-cause mortality in the entire cohort (HR per 1-SD, 1.82; 95% CI 1.60–2.07; Table 2). The other independent predictors included sex, long-term GRACE risk score, LVEF, BMI, history of diabetes, and acute use of aspirin, clopidogrel/ticagrelor, and statins (Table 2).

**Table 2:** Univariable and multivariable Cox regression analysis for all-cause mortality

Variables	Univariable model			Multivariable model		
	HR	95% CI	*P* value	HR	95% CI	*P* value
Female	1.81	1.56–2.10	< 0.001	0.81	0.68–0.96	0.016
Ln NT-proBNP, per 1-SD	3.46	3.14–3.80	< 0.001	1.82	1.60–2.07	< 0.001
Long-term GRACE risk score, per decile	1.56	1.51–1.62	< 0.001	1.31	1.25–1.37	< 0.001
LVEF, per 5%	0.78	0.75–0.80	< 0.001	0.95	0.92–0.99	0.011
BMI, per 1 kg/m^2^	0.87	0.86–0.89	< 0.001	0.97	0.95–0.99	0.015
Hypertension	1.51	1.28–1.78	< 0.001			
Diabetes mellitus	1.50	1.29–1.74	< 0.001	1.19	1.01–1.40	0.034
Ln hs-CRP, per unit	1.37	1.28–1.46	< 0.001			
Ln peak value of hs-TnI, per unit	0.98	0.95–1.02	0.354			
Aspirin	0.14	0.12–0.17	< 0.001	0.48	0.38–0.60	< 0.001
Clopidogrel/Ticagrelor	0.25	0.22–0.29	< 0.001	0.79	0.64–0.97	0.025
ACEI/ARB	0.45	0.39–0.53	< 0.001			
β-blocker	0.43	0.37–0.50	< 0.001			
Statins	0.23	0.20–0.27	< 0.001	0.65	0.52–0.81	< 0.001
Diuretics	2.52	2.13–2.99	< 0.001			

ACEI, angiotensin-converting enzyme inhibitor; ARB, angiotensin receptor blocker; BMI, body mass index; CI, confidence interval; GRACE, Global Registry of Acute Coronary Events; HR, hazard ratio; hs-CRP, high sensitivity C-reactive protein; hs-TnI, high sensitivity troponin I; LVEF, left ventricular ejection fraction; NT-proBNP, N-terminal pro-B-type natriuretic peptide

**Fig. 2:**
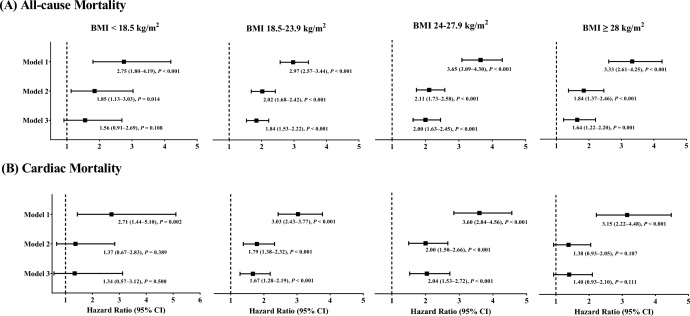
Forest plots of hazard ratios of Ln NT-proBNP per 1-SD for all-cause and cardiac mortality across the BMI categories. Model 1 was unadjusted. Model 2 was adjusted for long-term GRACE risk score, and LVEF. Model 3 was adjusted for covariates in model 2 plus sex, diabetes, aspirin, clopidogrel/ticagrelor, and statins

The associations between NT-proBNP concentration and the mortality risk across BMI categories are shown in Fig. 2. The multivariable adjusted HR (95% CI) for 1-SD increase in the ln-transformed NT-proBNP levels was 1.56 (0.91–2.69), 1.84 (1.53–2.22), 2.00 (1.63–2.45), and 1.64 (1.22–2.20) for the underweight, normal-weight, overweight and obese categories, respectively (Fig. 2a). Similar results were observed for cardiac mortality in most BMI categories, except for the underweight and obesity categories. (Fig. 2b).

Comparative analyses (NRI, IDI, and changes in the C-statistic) of the NT-proBNP levels against the long-term GRACE risk score as the reference model for each BMI category are shown in Table 3. The NT-proBNP levels significantly improved the accuracy beyond the long-term GRACE risk score alone to predict all-cause mortality in all BMI categories, except for the underweight category. A similar increment in prognostic accuracy of cardiac mortality was found for NT-proBNP levels in the normal-weight and overweight BMI categories (Additional file 4).

**Table 3:** Improvement in all-cause mortality risk prediction by adding NT-ProBNP to clinical models across the BMI categories

Clinical models	C-Statistics [95% CI]	△ in C-Statistics	*P* Value	NRI [95% CI]	IDI [95% CI]
BMI < 18.5 kg/m^2^					
Long-term GRACE risk score	0.709 [0.637–0.781]	Reference	Reference	Reference	Reference
NT-proBNP	0.722 [0.652–0.793]	0.014	0.710	0.062 [− 0.236–0.364]	− 0.010 [− 0.149–0.153]
Long-term GRACE risk score + NT-proBNP	0.747 [0.679–0.815]	0.038	0.124	0.270 [− 0.049–0.496]	0.066 [− 0.011–0.175]
BMI 18.5–23.9 kg/m^2^					
Long-term GRACE risk score	0.757 [0.727–0.786]	Reference	Reference	Reference	Reference
NT-proBNP	0.764 [0.735–0.794]	0.008	0.624	− 0.022 [− 0.135–0.112]	− 0.002 [− 0.058–0.057]
Long-term GRACE risk score + NT-proBNP	0.793 [0.765–0.821]	0.036	< 0.001	0.263 [0.177–0.358]	0.076 [0.043–0.116]
BMI 24–27.9 kg/m^2^					
Long-term GRACE risk score	0.807 [0.780–0.835]	Reference	Reference	Reference	Reference
NT-proBNP	0.808 [0.781–0.835]	0.001	0.949	− 0.072 [− 0.178–0.040]	− 0.036 [− 0.096–0.024]
Long-term GRACE risk score + NT-proBNP	0.849 [0.827–0.871]	0.042	< 0.001	0.204 [0.092–0.284]	0.046 [0.019–0.079]
BMI ≥ 28 kg/m^2^					
Long-term GRACE risk score	0.800 [0.752–0.847]	Reference	Reference	Reference	Reference
NT-proBNP	0.790 [0.748–0.832]	− 0.010	0.703	− 0.158 [− 0.316–0.032]	− 0.077 [− 0.156–− 0.002]
Long-term GRACE risk score + NT-proBNP	0.832 [0.791–0.872]	0.032	0.012	0.197 [0.063–0.340]	0.023 [0.000–0.065]

△ = difference

BMI, body mass index; CI, confidence interval; GRACE, Global Registry of Acute Coronary Events; IDI, integrated discrimination improvement; NRI, net reclassification index; NT-proBNP, N-terminal pro-B-type natriuretic peptide

**Fig. 3:**
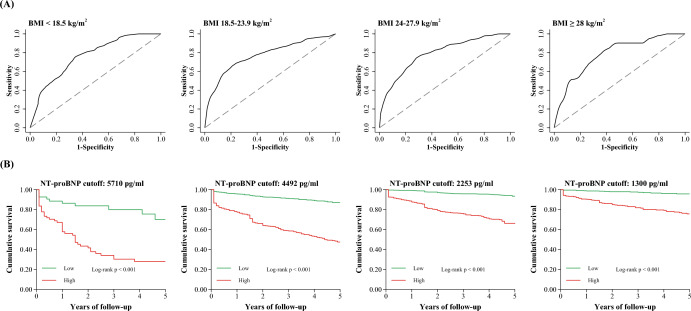
The ROC or Kaplan–Meier curves of NT-proBNP grouped by different BMI categories. The time-dependent receiver-operator curves of NT-proBNP for 5-year all-cause mortality **a** and Kaplan–Meier curves grouped by optimal NT-proBNP cutoffs **b** across the BMI categories

Time-dependent ROC analyses for each BMI category are shown in Fig. 3a. The best NT-proBNP cutoff values for predicting 5-year all-cause mortality risk decreased as the BMI increased (5710 pg/ml, 4492 pg/ml, 2253 pg/ml, and 1300 pg/ml for underweight, normal-weight, overweight, and obese, respectively; Table 4). A similar trend was observed for cardiac mortality (Table 4). Kaplan–Meier survival curves using the best NT-proBNP cutoff levels showed that higher NT-proBNP levels were markedly associated with an increased risk of all-cause and cardiac mortality in each BMI category (Fig. 3b and Additional file 5). Subgroup analyses revealed that the best NT-proBNP cutoff values varied among men and women (Additional file 6) and the young (< 65 years) and older (≥ 65 years) individuals (Additional file 7).

**Table 4:** The best NT-proBNP cutoff values in predicting 5-year all-cause and cardiac mortality across the BMI categories

	NT-proBNP cutoff (pg/ml)	95% CI	AUC	95% CI	Sensitivity	Specificity
5-year all-cause mortality						
BMI < 18.5 kg/m^2^	5710	1249–22026	0.752	0.619–0.859	0.754	0.654
BMI 18.5–23.9 kg/m^2^	4492	3678–6634	0.769	0.724–0.810	0.662	0.787
BMI 24–27.9 kg/m^2^	2253	1882–3328	0.789	0.752–0.825	0.747	0.715
BMI ≥ 28 kg/m^2^	1300	953–5271	0.781	0.728–0.830	0.822	0.618
5-year cardiac mortality						
BMI < 18.5 kg/m^2^	5710	1408–14618	0.660	0.535–0.782	0.810	0.541
BMI 18.5–23.9 kg/m^2^	3866	3361–6503	0.758	0.696–0.818	0.742	0.711
BMI 24–27.9 kg/m^2^	3294	1998–5014	0.793	0.739–0.844	0.710	0.759
BMI ≥ 28 kg/m^2^	1920	916–5486	0.770	0.691–0.841	0.761	0.677

AUC, area under curve; BMI, body mass index; CI confidence interval; NT-proBNP, N-terminal pro-B-type natriuretic peptide

The association between NT-proBNP concentrations and mortality risk did not substantially change in the sensitivity analyses using the WHO-defined BMI categories, except for the obesity group (BMI ≥ 30 kg/m^2^) (Additional file 8). Adding NT-proBNP levels significantly improved the accuracy beyond the long-term GRACE risk score alone to predict all-cause mortality in WHO-defined normal-weight and overweight categories (Additional file 9). The best NT-proBNP cutoff values for predicting 5-year mortality decreased as BMI increased using the WHO obesity criteria (Additional file 10).

## Discussion

In this large cohort study comprising more than 4600 patients with AMI, an inverse relationship between NT-proBNP levels and BMI was observed in the overall study population and for both sexes. We found that the NT-proBNP level was an independent prognostic factor for all-cause mortality in all BMI categories, except in underweight patients. Adding NT-proBNP to a conventional mortality risk model improved its risk discrimination and classification for each BMI category, except for the underweight group. Furthermore, the best NT-proBNP cutoff values for predicting mortality risk varied significantly across the BMI categories.

An inverse relationship between increasing BMI and NT-proBNP has been established in the general population and in those with heart failure [14, 15, 23, 24]. However, studies focusing on the AMI population, in whom the association between BMI and NT-proBNP levels may be more complex, are limited. Several determinants, such as the obesity paradox [12, 13] and reduced cardiomyocyte synthesis [25], may affect the prognostic value of NT-proBNP levels in AMI patients with different BMIs. The present study extended the investigation of the impact of BMI on NT-proBNP levels in patients after AMI.

Obesity is associated with cardiovascular diseases, with an increasing mortality rate [26–28]. Conversely, an apparent paradox of high BMI and reduced mortality has been observed in patients with cardiovascular disease [29–33]. Initially, the term ‘obesity paradox’ was coined to describe the improved prognosis in obese patients with systolic heart failure [29, 30]. The ‘obesity paradox’ has also been reported in patients with coronary heart disease and ACS. A meta-analysis of 40 cohort studies including 250,152 patients with coronary artery disease showed that overweight (BMI 25–29.9 kg/m^2^) patients had the lowest total (relative risk [RR]: 0.87; 95% CI 0.81–0.94) and cardiovascular (RR: 0.88; 95% CI 0.75–1.02) mortality risks compared with those with a normal BMI [34]. Another meta-analysis including 26 studies and 218,532 patients with ACS also confirmed that overweight (RR: 0.70; 95% CI 0.64–0.76), obese (RR: 0.60; 95% CI 0.53–0.68), and severely obese (RR: 0.70; 95% CI 0.58–0.86) patients had lower mortality compared with those with normal BMI [35]. The mechanism by which obese patients have better outcomes after AMI remains unclear. One potential explanation is that overweight and obese patients present earlier in the progression of symptoms of AMI, which may be related to increased awareness of their risk of having an AMI and earlier presentation and treatment [36]. Moreover, overweight and obese patients may also have less severe left ventricular dysfunction at presentation [36]. Further research is needed to determine the mechanisms underlying the ‘obesity paradox’ and its characteristics in certain patient subgroups.

In accordance with the favourable prognosis of high BMI in cardiovascular disease, significantly lower circulating NT-proBNP levels have been reported in overweight and obese patients [12–14]. One study of 618 patients with stable systolic congestive heart failure determined that BMI exerted a significant, independent, inverse influence on NT-proBNP levels, with a 4% drop in NT-proBNP level per unit increase in BMI, even after adjusting for cardiac function, age, sex, and renal function [12]. Similarly, a lower circulating NT-proBNP level in overweight and obese patients has been reported in an acute congestive heart failure population, which suggests a BMI-related defect in natriuretic peptide secretion [13]. A large prospective cohort study of 12,230 individuals in the general US population also indicated an inverse association between BMI and NT-proBNP levels (*β*-coefficient = − 0.10), which was maintained across race and sex subgroups [14]; these are consistent with our findings.

The potential underlying mechanisms by which low NT-proBNP levels may be associated with higher BMI remain unknown, with several hypotheses. First, reduced NT-proBNP secretion due to diminished myocardial release and impaired synthesis, rather than enhanced clearance, likely plays a larger role in patients with higher BMI [15, 37]. Furthermore, although the left ventricular end-diastolic pressure was elevated, obese patients had reduced NT-proBNP concentrations, which might indicate that determinants other than cardiac status affect the NT-proBNP levels [38]. Increased epicardial fat and heightened pericardial restraint might reportedly reduce wall stress and ventricular elaboration of natriuretic peptides [39]. Finally, sex and hormone status might also influence the circulating NT-proBNP concentrations, which might be associated with lower circulating androgen levels [40].

The predictive value of NT-proBNP levels has been well documented in patients with AMI. In most previous AMI studies [4–8], NT-proBNP was independently associated with an increased risk of mortality, even after adjusting for known important risk factors, such as age, heart rate, blood pressure, Killip class, and LVEF. However, BMI’s effect on the prognostic value of NT-proBNP in patients with AMI remained unclear. A French regional survey of 2217 individuals with AMI found that higher NT-proBNP levels were associated with increased 1-year cardiovascular mortality in normal and overweight patients when adjusted for the GRACE risk score and LVEF. However, NT-proBNP levels failed to retain their independent prognostic value in obese patients (OR = 1.34; 95% CI 0.86–2.08) [37]. We believe that such inconsistencies may be due to differences in the definitions of obesity. Asians have a higher amount of visceral fat at the same level of BMI than Caucasians [41]. Therefore, Chinese individuals may be at a higher risk of cardiovascular disease at the same level of obesity. This may explain why an increased NT-proBNP level was associated with a higher risk of mortality in obese patients using the Chinese criteria compared with that of the WHO criteria. On this basis, we further identified the best NT-proBNP cutoff values for predicting mortality risk according to the BMI classification for the Asian Pacific population. In this study, lower NT-proBNP cutoff levels were observed in overweight or obese patients than in those with normal weight, which might reclassify some of the patients as a high-risk population. As recommended by the latest European Society of Cardiology guidelines [42], using NT-proBNP levels to obtain more prognostic information should be considered in patients with non-persistent ST-segment elevation ACS (Class of Recommendation [COR]: IIa). This study highlights the impact of BMI on the optimal NT-proBNP cutoff values for risk prediction. However, this impact has not been considered in routine clinical practice. Furthermore, validated NT-proBNP cutoff values for patients with AMI are lacking, and its routine use for risk stratification or deciding the revascularization strategy is not yet recommended. Further prospective studies are required.

This study has several limitations that need to be addressed. First, this was a single-centre study in a Chinese population. It is important to note that the classification of obesity may vary across different populations. Therefore, caution should be exercised when generalising our findings. Second, only a single NT-proBNP measurement was used in this study, which might have introduced a potential bias due to measurement errors. Therefore, longitudinal analyses should be performed to confirm these findings. Third, obesity was defined by BMI, and more specific obesity-related indices, such as waist-to-hip ratio, body fat percentage, and visceral fat area, should be considered in further studies. Finally, information on the AMI severity or infarct size was not available in this study. Further external validation cohorts are warranted to explore the underlying mechanisms for the association between BMI and NT-proBNP levels and mortality in patients with AMI.

## Conclusion

The NT-proBNP level was an independent prognostic factor for mortality in patients with AMI. The best NT-proBNP cutoff values for predicting mortality risk varied across the BMI categories. Thus, the impact of BMI on this biomarker should be considered when predicting mortality risk in patients with AMI.

### Supplementary Information

**Additional file 1:** Study flow chart.

**Additional file 2:** NT-proBNP values in sex- and age-based subgroups across the BMI categories.

**Additional file 3:** Numbers of all-cause and cardiac death across the BMI categories.

**Additional file 4:** Improvement in cardiac mortality risk prediction by adding NT-ProBNP to clinical models across the BMI categories.

**Additional file 5:** The time-dependent receiver-operator curves of NT-proBNP for 5-year cardiac mortality (A) and Kaplan-Meier curves grouped by optimal NT-proBNP cutoffs (B) across the BMI categories.

**Additional file 6:** The best NT-proBNP cutoff values in predicting 5-year all-cause mortality across the BMI categories in either females or males.

**Additional file 7:** The best NT-proBNP cutoff values in predicting 5-year all-cause mortality across the BMI categories in patients < 65y or ≥ 65y.

**Additional file 8:** Cox regression analysis for the association of Ln NT-proBNP per 1-SD with all-cause and cardiac mortality across BMI categories defined by WHO.

**Additional file 9:** Improvement in all-cause mortality risk prediction by adding NT-proBNP to clinical models across the BMI categories defined by WHO.

**Additional file 10:** The best NT-proBNP cutoff values in predicting 5-year all-cause and cardiac mortality across the BMI categories defined by WHO.

## Data Availability

The data analyzed in this study can be obtained from the corresponding author with a reasonable request.

## Abbreviations

ACEI: Angiotensin-converting enzyme inhibitors
AMI: Acute myocardial infarction
ARB: Angiotensin II receptor blockers
BMI: Body mass index
CBDBANK: Cardiovascular Centre Beijing Friendship Hospital Database Bank
CI: Confidence interval
eGFR: Estimated glomerular filtration rate
FPG: Fasting plasma glucose
GRACE: Global Registry of Acute Coronary Events
HDL-C: High-density lipoprotein cholesterol
HR: Hazard ratio
hs-CRP: High-sensitivity C-reactive protein
hs-TnI: High-sensitivity Troponin I
IDI: Integrated discrimination improvement
LDL-C: Low-density lipoprotein cholesterol
LVEF: Left ventricular ejection fraction
MDRD: Modification of Diet in Renal Disease
NRI: Net reclassification improvement
NT-proBNP: N-terminal pro-B-type natriuretic peptide
PCI: Percutaneous coronary intervention
ROC: Receiver operator curve
