# Supplementary material for: Association of N-terminal pro-B-type natriuretic peptide levels and mortality risk in acute myocardial infarction across body mass index categories: an observational cohort study
Source: Diabetol Metab Syndr. 2023 Oct 6;15:192. doi: 10.1186/s13098-023-01163-1 (PMC10557200; doi:10.1186/s13098-023-01163-1)
Supplement: Supplementary file 1 — Additional file 1: Study flow chart. [file 13098_2023_1163_MOESM1_ESM.docx]

**Additional materials**

**Additional file 1.** Study flow chart.

**Additional file 2.** NT-proBNP values in sex- and age-based subgroups across the BMI categories.

**Additional file 3.** Numbers of all-cause and cardiac death across the BMI categories.

**Additional file 4.** Improvement in cardiac mortality risk prediction by adding NT-ProBNP to clinical models across the BMI categories.

**Additional file 5.** The time-dependent receiver-operator curves of NT-proBNP for 5-year cardiac mortality (A) and Kaplan-Meier curves grouped by optimal NT-proBNP cutoffs (B) across the BMI categories.

**Additional file 6.** The best NT-proBNP cutoff values in predicting 5-year all-cause mortality across the BMI categories in either females or males.

**Additional file 7.** The best NT-proBNP cutoff values in predicting 5-year all-cause mortality across the BMI categories in patients < 65y or ≥ 65y.

**Additional file 8.** Cox regression analysis for the association of Ln NT-proBNP per 1-SD with all-cause and cardiac mortality across BMI categories defined by WHO.

**Additional file 9.** Improvement in all-cause mortality risk prediction by adding NT-proBNP to clinical models across the BMI categories defined by WHO.

**Additional file 10.** The best NT-proBNP cutoff values in predicting 5-year all-cause and cardiac mortality across the BMI categories defined by WHO.


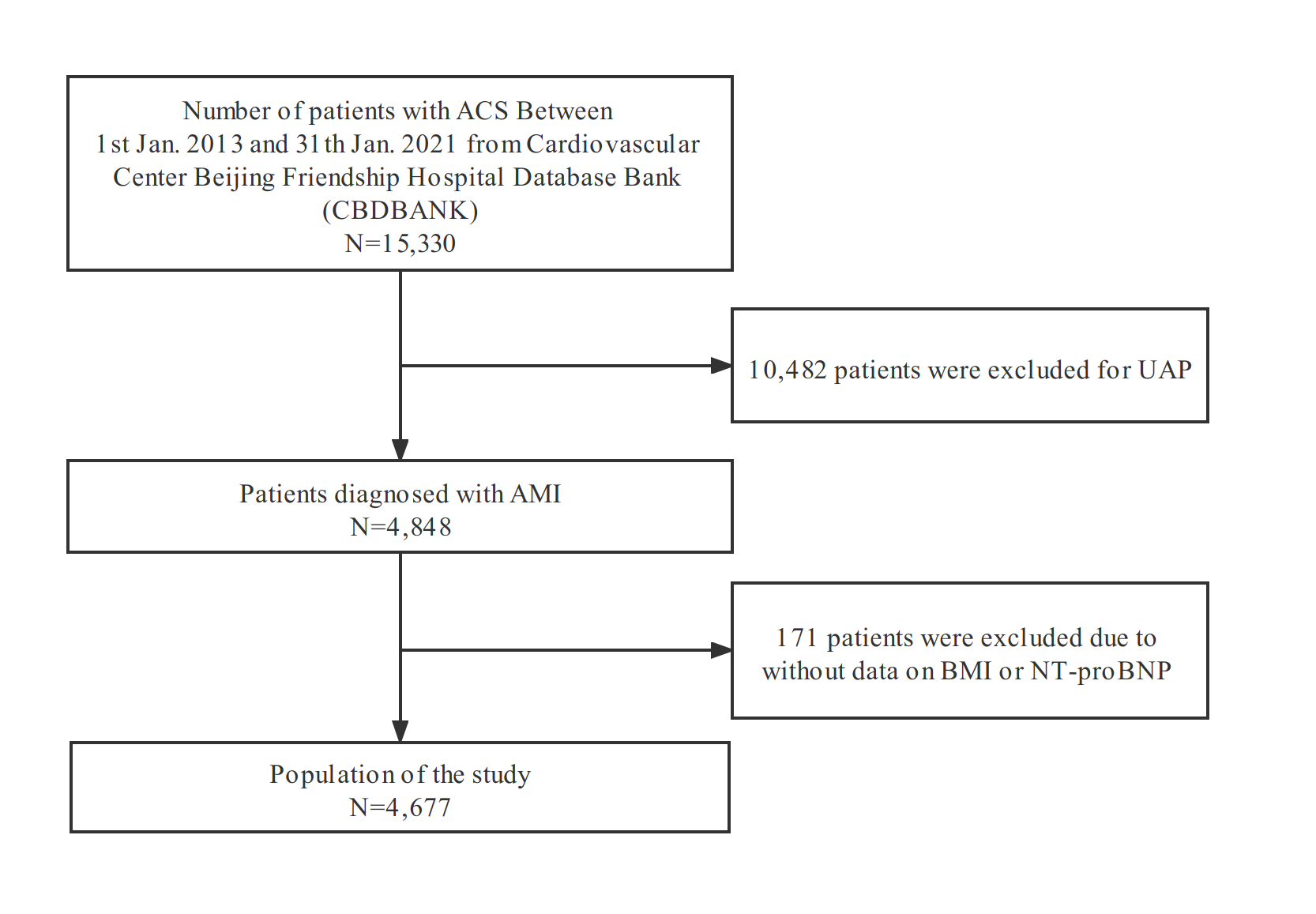


**Additional file 1. Study flow chart.**
