# Supplementary figures and images for: Association of N-terminal pro-B-type natriuretic peptide levels and mortality risk in acute myocardial infarction across body mass index categories: an observational cohort study
Source: Diabetol Metab Syndr. 2023 Oct 6;15:192. doi: 10.1186/s13098-023-01163-1 (PMC10557200; doi:10.1186/s13098-023-01163-1)

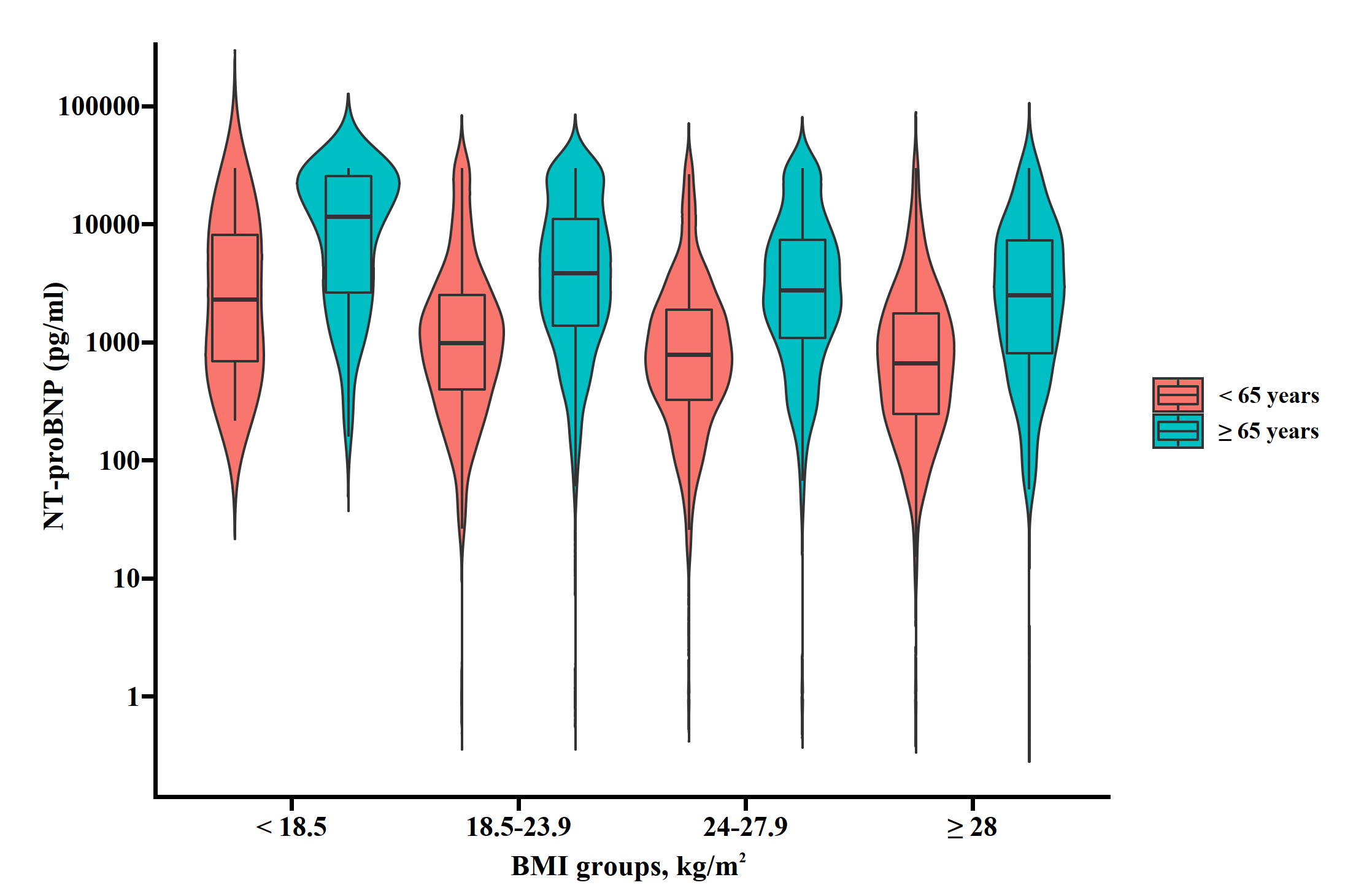

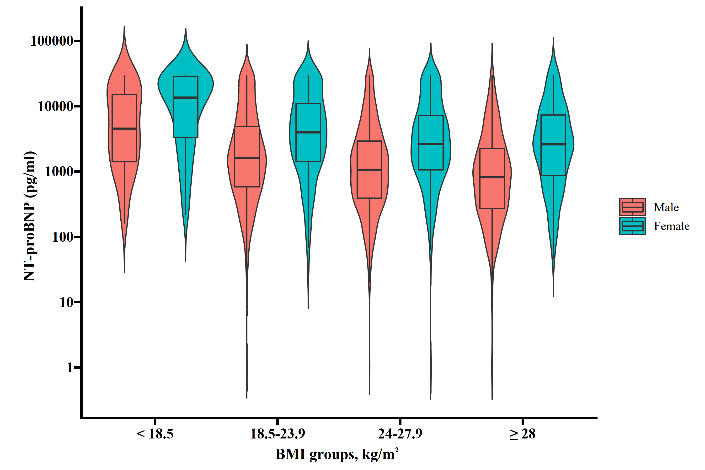
 **Additional file 2. NT-proBNP values in sex- and age-based subgroups across the BMI categories.**

Supplement: Supplementary file 2 — Additional file 2: NT-proBNP values in sex- and age-based subgroups across the BMI categories. [file 13098_2023_1163_MOESM2_ESM.docx]
