# Supplementary material for: Association of N-terminal pro-B-type natriuretic peptide levels and mortality risk in acute myocardial infarction across body mass index categories: an observational cohort study
Source: Diabetol Metab Syndr. 2023 Oct 6;15:192. doi: 10.1186/s13098-023-01163-1 (PMC10557200; doi:10.1186/s13098-023-01163-1)
Supplement: Supplementary file 3 — Additional file 3: Numbers of all-cause and cardiac death across the BMI categories. [file 13098_2023_1163_MOESM3_ESM.docx]

| **Additional file 3. Numbers of all-cause and cardiac death across the BMI categories.** | | | | | |
| --- | --- | --- | --- | --- | --- |
|  | **BMI < 18.5 kg/m^2^** | **BMI18.5**–**23.9 kg/m^2^** | **BMI 24**–**27.9 kg/m^2^** | **BMI ≥ 28 kg/m^2^** | ***P* value** |
| N, (%) | 127 (2.7) | 1496 (32.0) | 2029 (43.4) | 1025 (21.9) | - |
| All-cause death, n (%) | 58 (45.7) | 314 (21.0) | 243 (12.0) | 103 (10.0) | ＜0.001 |
| Cardiac death, n (%) | 25 (19.7) | 140 (9.4) | 117 (5.8) | 49 (4.8) | ＜0.001 |
| Abbreviations: BMI, body mass index. | | | | | |
