# Supplementary material for: Association of N-terminal pro-B-type natriuretic peptide levels and mortality risk in acute myocardial infarction across body mass index categories: an observational cohort study
Source: Diabetol Metab Syndr. 2023 Oct 6;15:192. doi: 10.1186/s13098-023-01163-1 (PMC10557200; doi:10.1186/s13098-023-01163-1)
Supplement: Supplementary file 4 — Additional file 4: Improvement in cardiac mortality risk prediction by adding NT-ProBNP to clinical models across the BMI categories. [file 13098_2023_1163_MOESM4_ESM.docx]

| **Additional file 4. Improvement in cardiac mortality risk prediction by adding NT-ProBNP to clinical models across the BMI categories.** | | | | | |
| --- | --- | --- | --- | --- | --- |
| **Clinical models** | **C-Statistics**  **[95% CI]** | **△ in C-Statistics** | ***P* Value** | **NRI [95% CI]** | **IDI [95% CI]** |
| BMI < 18.5 kg/m^2^ |  |  |  |  |  |
| Long-term GRACE risk score | 0.760 [0.686 to 0.833] | Reference | Reference | Reference | Reference |
| NT-proBNP | 0.704 [0.606 to 0.803] | -0.056 | 0.277 | -0.204 [-0.623 to 0.236] | -0.163 [-0.392 to 0.036] |
| Long-term GRACE risk score + NT-proBNP | 0.788 [0.712 to 0.864] | 0.028 | 0.206 | 0.252 [-0.254 to 0.510] | 0.012 [-0.047 to 0.106] |
| BMI 18.5–23.9 kg/m^2^ |  |  |  |  |  |
| Long-term GRACE risk score | 0.762 [0.719 to 0.805] | Reference | Reference | Reference | Reference |
| NT-proBNP | 0.758 [0.714 to 0.801] | -0.004 | 0.849 | -0.060 [-0.188 to 0.120] | -0.014 [-0.060 to 0.039] |
| Long-term GRACE risk score + NT-proBNP | 0.794 [0.753 to 0.835] | 0.032 | 0.006 | 0.249 [0.144 to 0.381] | 0.058 [0.026 to 0.099] |
| BMI 24–27.9 kg/m^2^ |  |  |  |  |  |
| Long-term GRACE risk score | 0.786 [0.748 to 0.825] | Reference | Reference | Reference | Reference |
| NT-proBNP | 0.791 [0.752 to 0.830] | 0.004 | 0.861 | -0.049 [-0.170 to 0.146] | -0.014 [-0.069 to 0.053] |
| Long-term GRACE risk score + NT-proBNP | 0.832 [0.801 to 0.863] | 0.046 | < 0.001 | 0.190 [0.069 to 0.308] | 0.031 [0.008 to 0.073] |
| BMI ≥ 28 kg/m^2^ |  |  |  |  |  |
| Long-term GRACE risk score | 0.842 [0.786 to 0.898] | Reference | Reference | Reference | Reference |
| NT-proBNP | 0.771 [0.713 to 0.828] | -0.071 | 0.045 | -0.279 [-0.476 to -0.034] | -0.106 [-0.190 to -0.028] |
| Long-term GRACE risk score + NT-proBNP | 0.855 [0.802 to 0.908] | 0.013 | 0.156 | 0.121 [-0.113 to 0.289] | 0.001 [-0.009 to 0.033] |
| △= difference.  BMI, body mass index; CI, confidence interval; GRACE, Global Registry of Acute Coronary Events; IDI, integrated discrimination improvement; NRI, net reclassification index; NT-proBNP, N-terminal pro-B-type natriuretic peptide. | | | | | |
