# Supplementary material for: Association of N-terminal pro-B-type natriuretic peptide levels and mortality risk in acute myocardial infarction across body mass index categories: an observational cohort study
Source: Diabetol Metab Syndr. 2023 Oct 6;15:192. doi: 10.1186/s13098-023-01163-1 (PMC10557200; doi:10.1186/s13098-023-01163-1)
Supplement: Supplementary file 5 — Additional file 5: The time-dependent receiver-operator curves of NT-proBNP for 5-year cardiac mortality (A) and Kaplan-Meier curves grouped by optimal NT-proBNP cutoffs (B) across the BMI categories. [file 13098_2023_1163_MOESM5_ESM.docx]

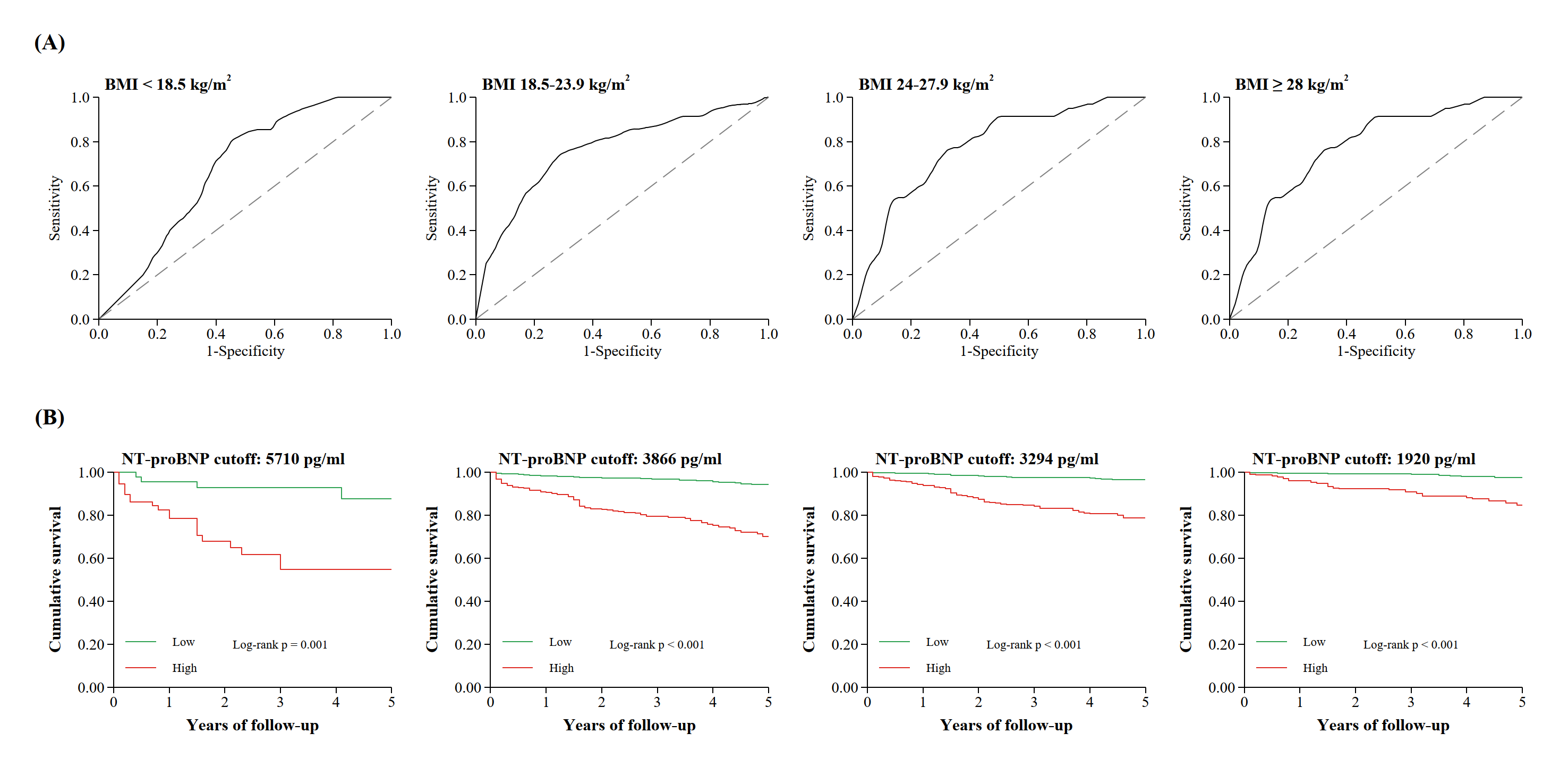


**Additional file 5. The time-dependent receiver-operator curves of NT-proBNP for 5-year cardiac mortality (A) and Kaplan-Meier curves grouped by optimal NT-proBNP cutoffs (B) across the BMI categories.**
