# Supplementary material for: Association of N-terminal pro-B-type natriuretic peptide levels and mortality risk in acute myocardial infarction across body mass index categories: an observational cohort study
Source: Diabetol Metab Syndr. 2023 Oct 6;15:192. doi: 10.1186/s13098-023-01163-1 (PMC10557200; doi:10.1186/s13098-023-01163-1)
Supplement: Supplementary file 6 — Additional file 6: The best NT-proBNP cutoff values in predicting 5-year all-cause mortality across the BMI categories in either females or males. [file 13098_2023_1163_MOESM6_ESM.docx]

| **Additional file 6. The best NT-proBNP cutoff values in predicting 5-year all-cause mortality across the BMI categories in either female or male.** | | | | | | | |
| --- | --- | --- | --- | --- | --- | --- | --- |
|  | | **NT-proBNP cutoff (pg/ml)** | **95% CI** | **AUC** | **95% CI** | **Sensitivity** | **Speciﬁcity** |
| **BMI < 18.5 kg/m^2^** | Female | 5541 | 1845–28001 | 0.667 | 0.476–0.820 | 0.818 | 0.498 |
|  | Male | 8519 | 1249–15678 | 0.837 | 0.686–0.942 | 0.629 | 0.854 |
| **BMI 18.5–23.9 kg/m^2^** | Female | 7332 | 3790–14472 | 0.765 | 0.711–0.820 | 0.632 | 0.781 |
|  | Male | 3752 | 3262–5825 | 0.761 | 0.704–0.816 | 0.657 | 0.818 |
| **BMI 24–27.9 kg/m^2^** | Female | 2208 | 1588–13095 | 0.786 | 0.717–0.842 | 0.882 | 0.548 |
|  | Male | 2039 | 1737–3395 | 0.787 | 0.735–0.831 | 0.719 | 0.737 |
| **BMI ≥ 28 kg/m^2^** | Female | 2298 | 1108–6836 | 0.726 | 0.634–0.814 | 0.815 | 0.544 |
|  | Male | 973 | 934–4537 | 0.764 | 0.688–0.831 | 0.850 | 0.596 |
| AUC, area under curve; BMI, body mass index; CI confidence interval; NT-proBNP, N-terminal pro-B-type natriuretic peptide. | | | | | | | |
