# Supplementary material for: Association of N-terminal pro-B-type natriuretic peptide levels and mortality risk in acute myocardial infarction across body mass index categories: an observational cohort study
Source: Diabetol Metab Syndr. 2023 Oct 6;15:192. doi: 10.1186/s13098-023-01163-1 (PMC10557200; doi:10.1186/s13098-023-01163-1)
Supplement: Supplementary file 7 — Additional file 7: The best NT-proBNP cutoff values in predicting 5-year all-cause mortality across the BMI categories in patients < 65y or ≥ 65y. [file 13098_2023_1163_MOESM7_ESM.docx]

| **Additional file 7. The best NT-proBNP cutoff values in predicting 5-year all-cause mortality across the BMI categories in patients < 65y or ≥ 65y.** | | | | | | | |
| --- | --- | --- | --- | --- | --- | --- | --- |
|  | | **NT-proBNP cutoff (pg/ml)** | **95% CI** | **AUC** | **95% CI** | **Sensitivity** | **Speciﬁcity** |
| **BMI < 18.5 kg/m^2^** | < 65y | 9414 | 880–10829 | 0.927 | 0.710–1.000 | 0.778 | 0.968 |
|  | ≥ 65y | 5597 | 1408–25084 | 0.697 | 0.540–0.821 | 0.762 | 0.598 |
| **BMI 18.5–23.9 kg/m^2^** | < 65y | 3134 | 1033–6701 | 0.693 | 0.567–0.797 | 0.519 | 0.834 |
|  | ≥ 65y | 6836 | 3905–7785 | 0.748 | 0.700–0.794 | 0.615 | 0.792 |
| **BMI 24–27.9 kg/m^2^** | < 65y | 2231 | 889–3328 | 0.750 | 0.660–0.835 | 0.613 | 0.816 |
|  | ≥ 65y | 2298 | 1863–10405 | 0.722 | 0.667–0.771 | 0.769 | 0.540 |
| **BMI ≥ 28 kg/m^2^** | < 65y | 1326 | 925–4024 | 0.723 | 0.611–0.843 | 0.696 | 0.701 |
|  | ≥ 65y | 2515 | 907–5653 | 0.703 | 0.631–0.769 | 0.725 | 0.595 |
| AUC, area under curve; BMI, body mass index; CI confidence interval; NT-proBNP, N-terminal pro-B-type natriuretic peptide. | | | | | | | |
