# Supplementary material for: Association of N-terminal pro-B-type natriuretic peptide levels and mortality risk in acute myocardial infarction across body mass index categories: an observational cohort study
Source: Diabetol Metab Syndr. 2023 Oct 6;15:192. doi: 10.1186/s13098-023-01163-1 (PMC10557200; doi:10.1186/s13098-023-01163-1)
Supplement: Supplementary file 8 — Additional file 8: Cox regression analysis for the association of Ln NT-proBNP per 1-SD with all-cause and cardiac mortality across BMI categories defined by WHO. [file 13098_2023_1163_MOESM8_ESM.docx]

| **Additional file 8. Cox regression analysis for the association of Ln NT-proBNP per 1-SD with all-cause and cardiac mortality across BMI categories defined by WHO.** | | | | | | | |
| --- | --- | --- | --- | --- | --- | --- | --- |
|  | **Model 1 ^a^** | | **Model 2 ^b^** | | **Model 3 ^c^** | | |
|  | **HR (95% CI)** | ***P* value** | **HR (95% CI)** | ***P* value** | **HR (95% CI)** | | ***P* value** |
| **All-cause mortality** | | | | | | | |
| BMI < 18.5 kg/m^2^ | 2.75 (1.80–4.19) | < 0.001 | 1.85 (1.13–3.03) | 0.014 | 1.56 (0.91–2.69) | 0.108 | |
| BMI 18.5–24.9 kg/m^2^ | 3.03 (2.66–3.45) | <0.001 | 1.98 (1.69–2.32) | <0.001 | 1.86 (1.59–2.19) | <0.001 | |
| BMI 25–29.9 kg/m^2^ | 3.94 (3.30–4.69) | <0.001 | 2.29 (1.84–2.85) | <0.001 | 2.11 (1.69–2.63) | <0.001 | |
| BMI ≥ 30 kg/m^2^ | 2.72 (1.91–3.86) | <0.001 | 1.47 (0.97–2.23) | 0.066 | 1.36 (0.90–2.07) | 0.149 | |
| **Cardiac mortality** |  |  |  |  |  |  | |
| BMI < 18.5 kg/m^2^ | 2.71 (1.44–5.10) | 0.002 | 1.37 (0.67–2.83) | 0.389 | 1.34 (0.57–3.12) | 0.500 | |
| BMI 18.5–24.9 kg/m^2^ | 3.03 (2.50–3.66) | <0.001 | 1.77 (1.41–2.21) | <0.001 | 1.69 (1.34–2.13) | <0.001 | |
| BMI 25–29.9 kg/m^2^ | 3.80 (2.95–4.89) | <0.001 | 2.04 (1.50–2.76) | <0.001 | 2.02 (1.49–2.75) | <0.001 | |
| BMI ≥ 30 kg/m^2^ | 2.50 (1.46–4.29) | 0.001 | 0.86 (0.47–1.58) | 0.630 | 1.00 (0.52–1.90) | 0.991 | |
| ^a^ unadjusted | | | | | | | |
| ^b^ Adjusted for Ln NT-proBNP, long-term GRACE risk score, and LVEF. | | | | | | | |
| ^c^ Adjusted for covariates in model 2 plus sex, diabetes, aspirin, clopidogrel/ticagrelor, and statins. | | | | | | | |
| Abbreviations: BMI, body mass index; CI, confidence interval; GRACE, Global Registry of Acute Coronary Events; HR, hazard ratio; NT-proBNP, N-terminal pro-B-type natriuretic peptide; WHO, World Health Organization. | | | | | | | |
