# Supplementary material for: Association of N-terminal pro-B-type natriuretic peptide levels and mortality risk in acute myocardial infarction across body mass index categories: an observational cohort study
Source: Diabetol Metab Syndr. 2023 Oct 6;15:192. doi: 10.1186/s13098-023-01163-1 (PMC10557200; doi:10.1186/s13098-023-01163-1)
Supplement: Supplementary file 9 — Additional file 9: Improvement in all-cause mortality risk prediction by adding NT-proBNP to clinical models across the BMI categories defined by WHO. [file 13098_2023_1163_MOESM9_ESM.docx]

| **Additional file 9. Improvement in all-cause mortality risk prediction by adding NT-proBNP to clinical models across the BMI categories defined by WHO.** | | | | | |
| --- | --- | --- | --- | --- | --- |
|  | **C-Statistics**  **[95% CI]** | **△ in C-Statistics** | ***P* Value** | **NRI [95% CI]** | **IDI [95% CI]** |
| **BMI < 18.5 kg/m^2^** |  |  |  |  |  |
| GRACE risk score | 0.709 [0.637 to 0.781] | Reference | Reference | Reference | Reference |
| GRACE risk score + NT-proBNP | 0.747 [0.679 to 0.815] | 0.038 | 0.112 | 0.270 [-0.007 to 0.514] | 0.066 [-0.006 to 0.176] |
| **BMI 18.5–24.9 kg/m^2^** |  |  |  |  |  |
| GRACE risk score | 0.768 [0.743 to 0.794] | Reference | Reference | Reference | Reference |
| GRACE risk score + NT-proBNP | 0.802 [0.779 to 0.826] | 0.034 | < 0.001 | 0.237 [0.157 to 0.310] | 0.064 [0.035 to 0.094] |
| **BMI 25–29.9 kg/m^2^** |  |  |  |  |  |
| GRACE risk score | 0.808 [0.778 to 0.838] | Reference | Reference | Reference | Reference |
| GRACE risk score + NT-proBNP | 0.856 [0.832 to 0.879] | 0.048 | < 0.001 | 0.232 [0.136 to 0.318] | 0.056 [0.025 to 0.092] |
| **BMI ≥ 30 kg/m^2^** |  |  |  |  |  |
| GRACE risk score | 0.804 [0.733 to 0.874] | Reference | Reference | Reference | Reference |
| GRACE risk score + NT-proBNP | 0.822 [0.758 to 0.885] | 0.018 | 0.254 | 0.152 [-0.119 to 0.357] | 0.012 [-0.006 to 0.055] |
| △= difference.  Abbreviations: BMI, body mass index; CI, confidence interval; IDI, integrated discrimination improvement; NRI, net reclassification index; NT-proBNP, N-terminal pro-B-type natriuretic peptide. | | | | | |
