# Supplementary material for: Association of N-terminal pro-B-type natriuretic peptide levels and mortality risk in acute myocardial infarction across body mass index categories: an observational cohort study
Source: Diabetol Metab Syndr. 2023 Oct 6;15:192. doi: 10.1186/s13098-023-01163-1 (PMC10557200; doi:10.1186/s13098-023-01163-1)
Supplement: Supplementary file 10 — Additional file 10: The best NT-proBNP cutoff values in predicting 5-year all-cause and cardiac mortality across the BMI categories defined by WHO. [file 13098_2023_1163_MOESM10_ESM.docx]

| **Additional file 10. The best NT-proBNP cutoff values in predicting 5-year all-cause and cardiac mortality across the BMI categories defined by WHO.** | | | | | |
| --- | --- | --- | --- | --- | --- |
|  | **NT-proBNP cutoff (pg/ml)** | **AUC** | **Sensitivity** | | **Speciﬁcity** |
| **5-year all-cause mortality** | | | | | |
| BMI < 18.5 kg/m^2^ | 5710 | 0.752 | | 0.754 | 0.654 |
| BMI 18.5–24.9 kg/m^2^ | 4537 | 0.767 | | 0.640 | 0.798 |
| BMI 25–29.9 kg/m^2^ | 2276 | 0.797 | | 0.734 | 0.739 |
| BMI ≥ 30 kg/m^2^ | 916 | 0.767 | | 0.900 | 0.534 |
| **5-year cardiac mortality** |  |  | |  |  |
| BMI < 18.5 kg/m^2^ | 5710 | 0.660 | | 0.810 | 0.541 |
| BMI 18.5–24.9 kg/m^2^ | 3866 | 0.765 | | 0.729 | 0.725 |
| BMI 25–29.9 kg/m^2^ | 2253 | 0.788 | | 0.761 | 0.703 |
| BMI ≥ 30 kg/m^2^ | 4964 | 0.785 | | 0.608 | 0.854 |
| Abbreviations: AUC, area under curve; BMI, body mass index; CI confidence interval; NT-proBNP, N-terminal pro-B-type natriuretic peptide. | | | | | |
